# Mechanisms behind the Madness: How Do Zombie-Making Fungal Entomopathogens Affect Host Behavior To Increase Transmission?

**DOI:** 10.1128/mBio.01872-21

**Published:** 2021-10-05

**Authors:** Charissa de Bekker, William C. Beckerson, Carolyn Elya

**Affiliations:** a Department of Biology, College of Sciences, University of Central Florida, Orlando, Florida, USA; b Genomics and Bioinformatics Cluster, University of Central Florida, Orlando, Florida, USA; c Department of Organismic and Evolutionary Biology, Harvard University, Cambridge, Massachusetts, USA; University of Texas Health Science Center at Houston

**Keywords:** animal behavioral change, coevolution, host specialization, effectors, Hypocreales, Entomophthorales

## Abstract

Transmission is a crucial step in all pathogen life cycles. As such, certain species have evolved complex traits that increase their chances to find and invade new hosts. Fungal species that hijack insect behaviors are evident examples. Many of these “zombie-making” entomopathogens cause their hosts to exhibit heightened activity, seek out elevated positions, and display body postures that promote spore dispersal, all with specific circadian timing. Answering how fungal entomopathogens manipulate their hosts will increase our understanding of molecular aspects underlying fungus-insect interactions, pathogen-host coevolution, and the regulation of animal behavior. It may also lead to the discovery of novel bioactive compounds, given that the fungi involved have traditionally been understudied. This minireview summarizes and discusses recent work on zombie-making fungi of the orders Hypocreales and Entomophthorales that has resulted in hypotheses regarding the mechanisms that drive fungal manipulation of insect behavior. We discuss mechanical processes, host chemical signaling pathways, and fungal secreted effectors proposed to be involved in establishing pathogen-adaptive behaviors. Additionally, we touch on effectors’ possible modes of action and how the convergent evolution of host manipulation could have given rise to the many parallels in observed behaviors across fungus-insect systems and beyond. However, the hypothesized mechanisms of behavior manipulation have yet to be proven. We, therefore, also suggest avenues of research that would move the field toward a more quantitative future.

## INTRODUCTION

Understanding a pathogen’s chain of transmission—how it enters a susceptible host, causes infection, and transmits afterward—is fundamental to the study of infectious disease (1). While adaptive evolution in any of these links leads to more fit pathogen populations, it is the selective pressures on transmission that have given rise to the intricate spore dispersal strategies of so-called “zombie-making” entomopathogenic fungi. Tiny insect hosts create a dual problem for transmission as they provide small amounts of carbon to sustain spore production and little surface area for spore structure presentation. The sheer biomass of epizootics, such as the large, seasonal outbreaks in house flies caused by Entomophthora muscae (2–7), could compensate for this. Moreover, certain entomopathogen species have optimized their dispersal by instigating host behaviors that promote direct contact with conspecifics or abiotic modes of spread. Indeed, *E. muscae*, like many other behavior-manipulating fungi, induces its fly hosts to summit and attach to positional vantage points that positively affect wind-mediated spore dispersal and to spread their wings to provide more surface for spore production (8). As such, both infection quantity (e.g., number of individuals infected) and quality (e.g., elevation and body posture of infected individuals) are seemingly important for effective transmission.

An estimated 1.5 million fungal species are entomopathogenic (9), demonstrating their substantial roles across ecosystems. Nevertheless, fungal entomopathogens have historically received less attention compared to their plant pathogen counterparts (10). This leaves much to be discovered about their modes of infection and transmission, natural products, and effector secretion during pathogen-host interaction. Beauveria (also *Cordyceps*) bassiana and several species of Metarhizium are among the relatively few fungal species that have been studied from a mechanistic perspective (11–13). These few characterized species represent generalist fungi that kill and consume their hosts in a matter of days upon infection without eliciting obvious pathogen-adaptive behavioral alterations. As such, one could consider these fungi to have a necrotrophic lifestyle. This is in contrast with behavior-manipulating species, which have a limited to highly specific host range and can spend more time in a symbiotic relationship with their insect host prior to killing it: a lifestyle that could be considered more hemibiotrophic, as a premature death of the host disrupts the fungal pathogen’s life cycle (14). Therefore, *Beauveria* and Metarhizium might not be the most appropriate species to draw parallels from when forming hypotheses about the molecular workings of manipulating pathogens.

Moreover, there are many other examples of insect-manipulating fungi that are not part of the order Hypocreales (Phylum: Ascomycota) like *Beauveria* and Metarhizium. Another large, diverse group of zombie-making fungi reside in the order Entomophthorales (Phylum: Zoopagomycota). Major fundamental differences between the life cycles and structures of Ascomycota and Zoopagomycota likely impact the molecular underpinnings of host manipulation. As such, our knowledge of the mechanisms that drive fungus-adaptive behavioral phenotypes is limited, especially with regard to the less lab-amenable Entomophthorales species (15). Despite these challenges, mycologists have begun to expand fungal biology, ecology, and evolution studies to include more obscure entomopathogenic species and explore their genomes, transcriptomes, proteomes, and metabolomes in recent years (16–22). This vastly growing body of work has led to various hypotheses about the mechanistic underpinnings that these fungi might employ to manipulate their insect hosts. Since host manipulation is a trait that has evolved multiple times independently (23, 24), the question arises whether zombie-making fungi could have evolved comparable mechanisms to infect and manipulate their hosts. In this review, we discuss the mechanistic hypotheses that have been posed based on manipulating-fungus research across the spectrum (Table 1). As such, we review previously reported studies of behavior-manipulating Hypocreales and Entomophthorales species, compare data to investigate if there are parallels that would suggest the evolution of comparable host manipulation mechanisms, and propose next steps to test those hypotheses.

**TABLE 1:** Observed behavior manipulations by entomopathogenic fungi with their proposed fungal benefit, hypothesized underlying fungal mechanisms, and potential host pathways of action

Induced behavior	Fungal benefit	Fungal mechanism	Host pathway(s)	Example fungi (hosts)	Example references
Time-specific behaviors	Aligns fungal emergence with favorable abiotic factors	Effector secretion, disruption of sensory periphery	Biological clock	*Ophiocordyceps* spp. (*Camponotus*); *Entomophthora muscae* (Musca domestica, *Drosophila*)	17, 28, 36, 47, 48, 77
Light seeking	Positions host in favorable microenvironment	Effector secretion	Biological clock, phototaxis	*Ophiocordyceps* spp. (*Camponotus*, *Colobopsis*)	32, 33
Hyperactivity	Avoidance of social immunity, facilitates summiting	Effector secretion, host nutrient depletion	Locomotion, arousal, hunger	*Ophiocordyceps* spp. (*Camponotus*)	21, 28
Summit disease	Increases spore dispersal	Effector secretion	Thermotaxis, phototaxis, gravitaxis	*Entomophthora muscae* (Musca domestica, *Drosophila*); *Entomophaga grylli* (*Melanoplus bivittatus*); *Ophiocordyceps* spp. (*Camponotus*, *Colobopsis*); *Eryniopsis lampyridarum* (*Chauliognathus pensylvanicus*)	8, 35, 36, 39, 49
Surface adherence	Prevents falling from vantage points that increase spore dispersal	Hydrophobic protein secretion, growth in/around mandibular muscle, hyphal anchoring	Proboscis; mandibles and legs	*Entomophthora muscae* (Musca domestica, *Drosophila*); *Pandora* (*Formica*), *Ophiocordyceps* spp. (*Camponotus*)	8, 17, 30, 36, 58, 121
Splayed wings	Removes barriers for spore dispersal	Growth patterns in/around thoracic muscle	Intrathoracic pressure	*Entomophthora muscae* (Musca domestica, *Drosophila*); *Eryniopsis lampyridarum* (*Chauliognathus pensylvanicus*)	8, 17, 36, 49
Increased sexual behavior	Increases transmission via direct contact	Effector secretion	Sexual arousal, locomotion	*Massospora* (*Cicada*)	19, 25

## MANIPULATED INSECT BEHAVIORS: PARALLELS ACROSS FUNGI

Manipulated insect behaviors increase fungal fitness by making spore dispersal more effective, either prior to or after host death. *Massospora*, for example, produces infective spores while keeping its cicada hosts alive, coercing them to actively transmit the infection to conspecifics (25). Success of *Massospora* spp. depends on their ability to consume the host and rupture its abdomen to produce spores, while keeping the insect intact enough to remain active and facilitate dispersal. Behavior-manipulating fungi that kill their insect hosts prior to spore production are not so different in their approach to increase transmission. Many entomophthoralean and hypocrealean fungi rely on host movement to transport them toward conditions that favor spore production and dissemination. Fungal manipulators that infect eusocial insects (i.e., *Pandora* and *Ophiocordyceps* species) appear to particularly benefit from increased locomotion activities in their hosts (21, 26–28). *Ophiocordyceps*-infected ants display a directionless, constant locomotion activity that hampers effective foraging efforts (28). Such wandering behavior could cause infected individuals to stray from the ant colony before the infection is noticed by nest mates. This is likely essential for fungal survival as healthy ants attack infected individuals as part of their social immunity strategy, which interferes with eventual spore formation and transmission (21, 28). Therefore, host activity can also be viewed as a spore dispersal strategy used by fungi that transmit after host death.

Hyperlocomotion and wandering likely also aid in establishing the next manipulation step that is often induced by fungi that kill their hosts prior to transmission: summiting. Many manipulating Entomophthorales and Hypocreales species coerce their insect hosts to ascend vegetation toward the end of the infection (8, 29). Ejecting spores from an elevated position is thought to increase transmission through more effective wind dispersal (30, 31). The upward locomotory movement may be accompanied by “light-seeking behavior,” as suggested for Ophiocordyceps camponoti-atricipis and Ophiocordyceps pseudolloydii (32, 33). Additionally, cooler-temperature seeking by *E. muscae* has been observed during late stages of infection, though it is not yet known if this behavior is a fungal manipulation or a general response to sickness (34). Overall, inducing a preference for specific abiotic factors could increase the likelihood that insect hosts die at elevated positions, serving to distance them from aggressive conspecifics as well as facilitate the most optimal microhabitat (i.e., temperatures, humidity, and light levels) for fungal growth (30, 32). Indeed, experiments that manipulated incipient light levels or removed *Ophiocordyceps*-infected cadavers from their original positions largely disrupted fungal fruiting body development (14, 32, 35).

Once the hijacked host has arrived at its final destination, fungal pathogens that cause summiting often induce one final behavior to ensure substrate adherence. *Entomophthora* species that infect dipterans cause them to attach to vegetation by extending their proboscis (17, 36), while *Pandora* and *Ophiocordyceps* species induce biting behavior in their hymenopteran hosts (30, 37). Depending on the substrate type and fungal species involved, this behavior can be accompanied by the insect folding its legs around the vegetation for additional support with the fungus sometimes further securing the position by “fixing” the cadaver with hyphae (16, 23, 33, 36, 38, 39). Additionally, *Entomophthora*-infected insects often extend their wings out of the line of fire for ejected spores, providing a clear path to new hosts (17, 36).

Crucially, this final cascade of manipulated behaviors does not take place at just any time of day. Studies noting the timing of manipulated behaviors for particular fungus-insect interactions, as well as other infectious agents beyond the fungal kingdom (40–46), indicate that they occur with a certain daily timing. *Ophiocordyceps*-infected ants displayed biting behavior around solar noon (35), likely to facilitate light-seeking behavior (32, 47, 48), Eryniopsis lampyridarum-infected soldier beetles die in the morning (49), while *Entomophthora*-infected flies die at dusk (17, 36). As such, the manipulation of daily timing in the host appears to be an additional strategy that can be found across the board.

**FIG 1:**
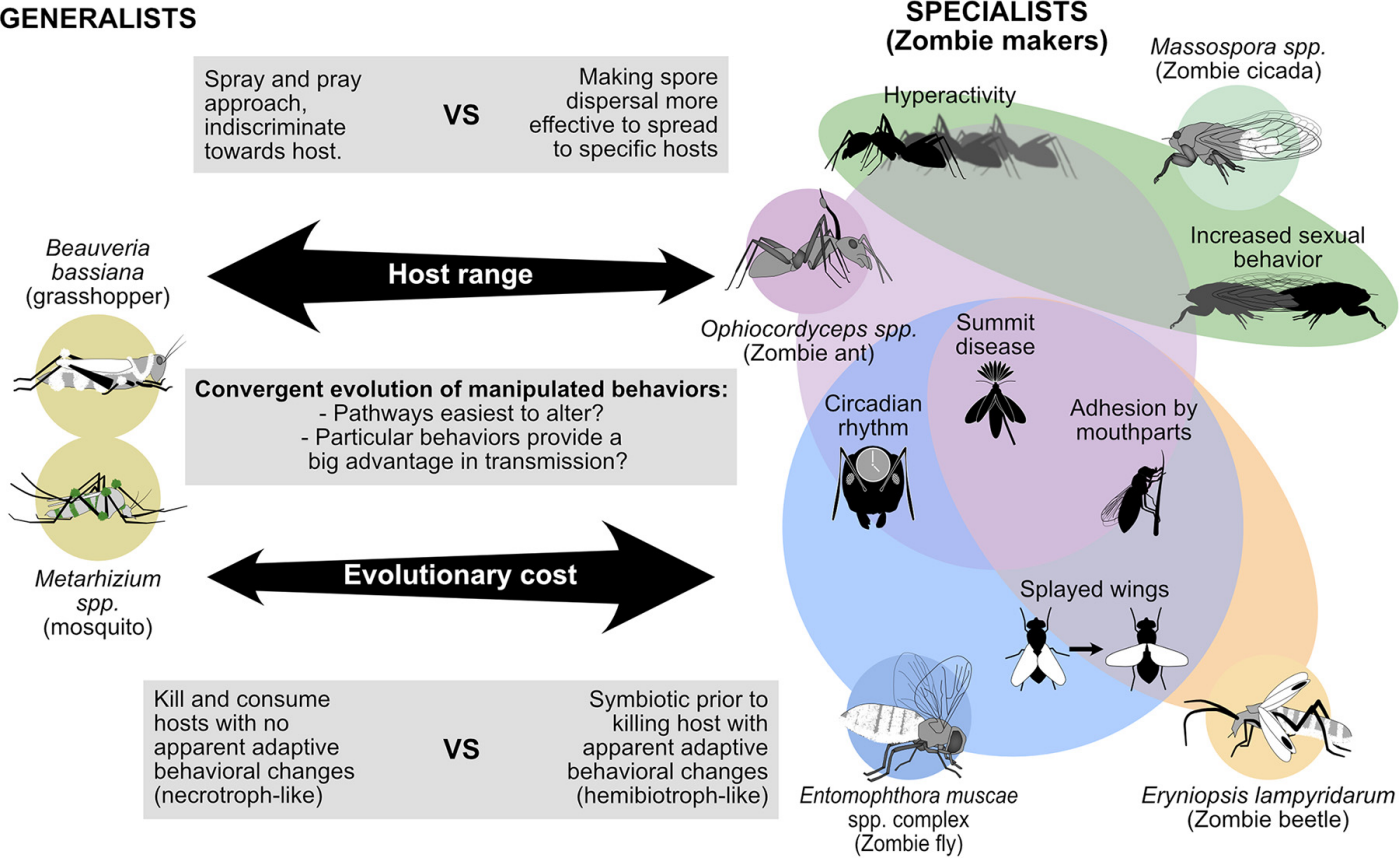
Differences between generalist and specialist, zombie-making fungal entomopathogens and the overlap between manipulated behaviors observed across specialist pathogen-host systems.

Fungi that independently evolved behavior-hijacking strategies to enhance transmission cause manipulated behaviors with several parallels across fungus-insect interactions (46, 50–52) (Fig. 1). Notably, ascension behaviors are also induced by nonfungal pathogens and parasites, such as baculoviruses and trematodes (38–40, 53). For one baculovirus strain, two virus genes (i.e., *ptp* and *egt*) are seemingly involved in causing enhanced locomotion and climbing behavior in its caterpillar host (54, 55). Additionally, histology work on trematode species that infect ants showed that the parasite’s physical positioning relative to host nervous tissues determines if climbing is accompanied by biting behavior (53, 56, 57). Behavioral parallels between these zombie-makers and fungi make it plausible that comparable strategies to hijack host behaviors have evolved.

## POSSIBLE MECHANISMS

The endoparasitic life cycle of entomopathogenic fungi is intertwined with host tissue infiltration and consumption. Since zombie-making fungi rely on able-bodied hosts to execute fungus-adaptive, manipulated behaviors, they are likely to interact with host tissues in a more careful approach than a simple consume-all strategy. The delicate relationship between fungal cells and host tissues might have led to the evolution of successful host infiltration strategies that ultimately give rise to altered behaviors. Such strategies can be lumped into two major categories: chemical and mechanical processes. While chemical means of host manipulation include a wide breadth of secreted biomolecules that disrupt and otherwise manipulate the host cellular machinery at the molecular level, mechanical methods of manipulation involve broader physical changes to the host, e.g., tissue-specific infiltration and destruction, changes in internal pressures, and spore production in particular compartments.

### Mechanical processes.

Entomopathogenic fungi vary in which host tissues they occupy, nervous tissue being a prime example. There is mounting evidence that entomophthoralean species (*E. muscae* and Entomophthora aphidis, Entomophaga grylli, Strongwellsea castrans, Conidiobolus coronatus, Pandora formicae) infiltrate the brain tissue of their hosts (fruit flies, aphids, grasshoppers, anthomyiid flies, mosquitoes, and ants, respectively) while the hosts are still alive (17, 26, 58–62). In contrast, *Ophiocordyceps* species do not infiltrate the nervous tissue of their carpenter ant hosts until after death, despite their unabated expansion into the muscle tissue (35, 63, 64). Additionally, these two groups differ in the rate of disease progression. *Ophiocordyceps* infections take place slowly, over the course of weeks, providing more time for manipulation of behaviors that drive the ant away from the nest and upward (18, 21, 27). *Pandora*, on the other hand, infiltrates its ant hosts in a matter of days, resulting in mound biting or gripping onto blades of grass near the ant mound (26). The rate of pathogenicity and the rate of fungal expansion into various host tissues are, therefore, likely tied to selective pressures requiring differing degrees of host manipulation.

Another notable divergence between host-manipulating fungi is observed in the physical manipulation of the host immediately perimortem or postmortem. Summiting behavior, for example, favors a method to anchor the host to its substrate to provide enough time to produce spore-bearing structures without falling from its optimal spore dispersal vantage point. Adherence of the host is in many species accomplished via induction of biting behaviors collectively called the “death grip.” The mechanisms behind the death grip are still being explored and likely involve a combination of mechanical and chemical means. The death grip behavior in *Ophiocordyceps*-infected hosts may be, in part, caused by overgrowth of fungal tissue in the legs and mandibles (64). The fungus forms interconnected cells around individual mandibular muscle cells, occupying ∼40% of the biomass and causing muscle fibers to become widely separated. Separation of the muscle tissues causes Z-lines to become swollen, sarcomeres to shorten, and muscle fiber sarcolemma to degrade. In addition, hyphal bodies and extracellular vesicle-like particles were in direct contact with mandibular muscles, indicating that compound secretion may also play a role in muscle contraction (64). Mechanical means of adherence are likely facilitated by secretory compounds in other species as well. In hosts without mandibles, such as fruit flies infected by *E. muscae*, the proboscis is extended toward a substrate in a mechanical manner unlike the extension observed during eating. This proboscis extension is paired with the production of sticky secretions that are yet uncharacterized but hypothesized to be of fungal origin (17, 58).

Once the host is fixed to a surface, the fungus can emerge and disseminate spores. To make way for emerging conidiophores that carry the primary conidia in winged hosts, mechanical manipulation of the wings lifts them up and away from the dorsal abdomen (17). The raising of the wings observed in *E. muscae*-infected *Drosophila* happens over approximately 15 min and is thought to be caused by an increase in pressure against thoracic muscle (17). By raising the wings off the back, conidia can be forcibly ejected without being blocked (17). This splayed wing phenotype has also been studied in the soldier beetle Chauliognathus pensylvanicus infected with *E. lampyridarum*; however, *Eryniopsis*-induced wing raising takes much longer, occurring over the course of several hours (49). In addition to applying physical pressure to host tissue, entomopathogenic fungi consume and, thus, destroy it through the secretion of proteases, lipases, and chitinases (16, 22, 27, 65). This results in physical stress by impairing tissue integrity and in chemical stress by removing cells that produce signaling molecules involved in physiological homeostasis, either of which could trigger behavioral responses in the host. However, it seems unlikely that tissue destruction itself accounts for modified host behaviors that are pathogen adaptive, given that generalist entomopathogens like *Beauveria* and Metarhizium also destroy tissues in their living hosts and do not evoke the same, precise, behavioral changes.

While many mechanical host manipulations seem to be conserved across behavior-modifying entomopathogens, unique examples also exist. Massospora cicadina, a fungus that infects cicadas, causes destruction of the terminal abdomen where the genitalia of the host are located and replaces it with a fungal spore mass (25). In addition to removing the reproductive organs of the host to make way for infective conidiospores, *Massospora* species seem to hijack host reproductive behavior to spread more rapidly via direct contact. This strategy is likely facilitated by the secretion of compounds that increase the host’s desire to mate (25). Fungal overgrowth has also been implicated in the dysmotility observed in the antennae of *Ophiocordyceps*-infected ants. The antennae of Ophiocordyceps camponoti-floridani-infected Camponotus floridanus are commonly observed to be locked in a bent L-shaped position late in infection (28). While antennal movements are important for communication with nest mates and navigation, their obstruction likely inhibits these behaviors and may therefore contribute to the wandering behavior that drives the ant away from the nest.

While mechanical manipulations are seen across the spectrum of zombie-making fungi, their degree of influence throughout the infection process is still being explored. Studies involving scanning and transmission electron microscopy on the mandibular muscles of *Ophiocordyceps*-infected hosts are broadening our understanding of how these fungi position themselves within the host at the point of behavior manipulation (64). Further studies into the physical manipulation of hosts by host-specific behavioral manipulators and more generalist nonmanipulators may provide further insight into the elaborate colonization strategies that have evolved in zombie-making fungi. While it is clear that physical manipulation of the host is vital for completion of the fungal life cycle, many, if not all, of these forced behaviors are facilitated in part via chemical signaling between these pathogens and the neuromuscular systems of their hosts. Understanding the collaboration between physical and chemical means of host manipulation is necessary to understand how these pathogen-driven modifications have evolved.

### Chemical signaling.

Chemical signaling could be either directly or indirectly neuromodulatory: secreted fungal factors could act directly on the neural circuits underlying a given behavior or they could alter upstream inputs to these circuits, thereby triggering pathways that ultimately result in changed behavioral output. As mentioned, zombie-making fungi differ with respect to invasion of nervous tissue: hypocrealean fungi do not invade while entomophthoraleans do. This suggests that the two major clades of zombie-making fungi may employ complementary strategies to achieve broader behavioral changes. For Entomophthorales species, direct access to host neuropil may indicate a more direct approach to altering circuits underlying behavioral changes (i.e., secreted factors could act directly on neurons to change behavioral output). Lack of such direct access in hypocrealean fungi may indicate that behavior is altered through indirect routes (i.e., modulating internal state or integrity of tissue). Alternatively, the position of fungal cells relative to host nervous tissue may be dispensable for behavior alteration (i.e., both direct and indirect chemical signaling are possible mechanisms for behavior-manipulating fungi, regardless of their clade and mode of mechanical interaction).

Fungi could employ chemical signaling to alter host behavior via depletion of host nutritional reserves or via modulating host physiology/neurobiology through the release of chemical effectors. Depletion of host nutritional reserves resulting from consumption by the fungal pathogen can induce a starvation state that ultimately leads to host behavior changes as it seeks to replenish its stores. Transcriptomic data provide ample evidence of host starvation: in *Ophiocordyceps*-manipulated ants, the expression levels of genes implicated in response to starvation were found to be altered (e.g., lipase, amylase, insulin, juvenile-hormone-responsive cytochrome p450), while in *E. muscae*-infected fruit flies, metabolism Gene Ontology (GO) terms were significantly enriched among downregulated genes during late infection (17, 21). Moreover, increased locomotor activity as a result of acute starvation has been reported in several insects (66–69) and is reminiscent of the activity that insects demonstrate before they are killed by fungal pathogens (21, 28). However, nutrient depletion alone seems unlikely to account for the moribund behaviors of zombie fungus-infected insects since starving insects do not manifest summiting behavior or distinct postural changes. Given the tight temporal coupling of host resource depletion and manipulated behaviors, it will be important to tease out the role that starvation plays in driving observed end-of-life behaviors (i.e., how much does shifting internal state contribute to behavior manipulation). Studies using genetic tools or pharmaceuticals to prevent the host from sensing starvation or studies ectopically inducing satiety in nutrient-depleted hosts could be a reasonable starting point for this work.

Entomopathogenic fungi could also indirectly trigger behavioral responses through effectors, which we define as any molecule (e.g., metabolite, protein, or other biopolymer) that impacts host physiology. There is mounting evidence for the importance of protein effectors in zombie ants: transcriptomic analyses of two distinct *Ophiocordyceps*-ant interactions (i.e., Ophiocordyceps kimflemingiae-infected Camponotus castaneus and O. camponoti-floridani-infected *C. floridanus*) have revealed a putative enterotoxin to be highly expressed during manipulated summiting and biting behavior (27). While entomopathogenic fungi contain several enterotoxin-encoding genes in their genomes (with *Ophiocordyceps* species so far having been found to have the most), comparative genomic analysis found that orthologs of this specific enterotoxin are seemingly exclusively conserved among ant-manipulating *Ophiocordyceps* species (18). This suggests that this compound plays an important role in altering host ant behavior (21).

In addition, genomics and transcriptomics analyses have identified a large repertoire of small secreted proteins (SSPs) encoded by *Ophiocordyceps* spp. of which a significant number are upregulated during manipulated biting behavior (18, 21, 27). This suggests that SSPs may be promising candidates for behavior modifications. Many of these predicted SSPs have unknown functions since they cannot be classified based on known Pfam domains or GO terms. Additionally, they are often highly species specific, complicating homology-based annotations (21, 27). As such, SSPs comprise an interesting class of bioactive molecules that warrant functional investigations, not only because they could be key to the mechanisms underlying behavioral manipulation but also because they may result in the discovery of novel compounds that have medicinal or pest control applications.

Metabolite effectors have also been implicated in zombie behaviors. A recent study identified the presence of the alkaloids psilocybin (the active ingredient in hallucinogenic magic mushrooms) and amphetamine cathinone in Massospora levispora*-* (synonymous with Massospora platypediae [70]) and *M. cicadina*-infected cicadas (19). Analyses of genome sequences for these fungi revealed homologues for some of the genes known to be involved in synthesizing these alkaloids, while others were conspicuously absent. This led to the speculation that *Massospora* might possess novel means of biosynthesizing these compounds. Psilocybin and cathinone, like many other alkaloids, have well-known behavioral effects. This makes it plausible that these compounds are involved in the increased activity and hypersexuality behaviors observed in *Massospora*-infected cicadas (25). Additionally, *O. kimflemingiae* and *O. camponoti-floridani* contain at least one alkaloid-producing metabolite cluster that is predicted to be an aflatrem-like compound. The cluster was highly expressed during manipulation (21, 27), further suggesting that metabolite effectors may play important roles during behavior manipulation. However, several Metarhizium species have been shown to produce ergot alkaloids in live insect hosts but not in dead insects or on artificial media. This suggests a role for ergot alkaloids in insect colonization (71). Thus, though these findings are compelling, we cannot exclude the possibility that the role of these ergot alkaloids is restricted to killing or colonizing the ant host rather than driving behavior manipulation. Studies using fungal mutant strains for the genes that give rise to these compounds, as well as experiments testing the behavioral effects of these compounds in uninfected animals, will be helpful to determine the role they play in behavior manipulation (19). Beyond alkaloids, other metabolite effectors have also been implicated in *Ophiocordyceps*-ant brain interactions. In one study, brains of four different ant species, of which one was the naturally infected and manipulated host, were cocultured *ex vivo* with *O. kimflemingiae* and liquid chromatography-tandem mass spectrometry (LC-MS/MS) analysis was performed to detect the presence of secreted compounds (72). Resultant analysis found that *O. kimflemingiae* secreted a specific set of metabolites depending on the species of ant brain it encountered. Additionally, two compounds with potential neurobiological function, guanidinobutyric acid (GBA) and sphingosine, were found to be uniquely present in cocultures with the brain of the natural host, *C. castaneus* (72). A subsequent study looking at the metabolites in brains of *O. kimflemingiae*-infected *C. castaneus* confirmed that infection has a significant impact on brain metabolism even though the fungus does not physically contact the brain during manipulation (20). The study also detected a dramatic increase of ergothioneine in the brains of *O. kimflemingiae*-manipulated hosts compared to those of healthy hosts and hosts dying from infection by the non-behavior-modulating generalist B. bassiana. The authors of this study suggest that fungus-derived ergothioneine could be preserving nervous tissue during fungal infection (20).

It is not yet clear which putative effector genes can be implicated in behavior manipulation by entomophthoralean fungi. Both a paucity of genomic data (owing to extremely large, repeat-rich, and assembly-averse genomes) and their much greater phylogenetic distance from well-studied Ascomycota (often impeding inference of function by homology) mean we currently know much less about the genes expressed by these fungi over the course of infection and during manipulation. The predominant hypothesis in the field is that entomophthoralean fungi refrain from producing effectors to elude immune detection from their host, as well as to avoid premature host death (73, 74), but this should be reevaluated as more information becomes available.

### Possible sites of action.

Based on altered behavioral phenotypes, several pathways stand out as potential targets for fungal effectors (Table 1). Given the consistent circadian timing of “zombified” insect behaviors, one system likely targeted by fungal effectors is the host circadian clock. Work in *E. muscae* has found that house flies infected and housed in complete darkness (i.e., free-running conditions) die without consistent timing (36), even though healthy house flies maintain rhythmicity under free-running conditions (75). In contrast, flies infected and housed under a light-dark cycle for 72 h (i.e., entrainment conditions) prior to housing in darkness do demonstrate synchronized timing of death (36). The most parsimonious explanation for this observation is that the host clock does not drive timing of moribund behaviors and death. These events follow another clock, which requires Zeitgeber cues during early infection. Transcriptomic data have revealed that *E. muscae* expresses a homologue of *white-collar 1* (17), a photoreceptor and core component of the molecular clock in Neurospora crassa (76). This raises the possibility that the alternative clock that drives moribund behaviors could belong to *E. muscae* (17). In a similar vein, the *O. kimflemingiae* genome contains several homologues of N. crassa clock components, which are expressed in a circadian manner and drive the daily expression of genes annotated to be involved in pathogen-host interactions (77). Moreover, some of the host clock genes appear to be dysregulated in ants that display manipulated biting behavior (21, 27), suggesting that the fungal clock is also at play in the zombie ant system. In addition, or alternative to clock-regulated fungal effectors, changes in host timing may result from diminished sensing of Zeitgeber cues due to fungal cell growth and tissue integrity loss, which could lead to phase shifts and amplitude changes of host daily rhythms. Future research that bridges chronobiology and pathogen-host interactions (78) should address this possibility.

Pathways that control locomotion also seem likely targets of manipulation by fungal effectors. For example, zombie ants show increased locomotion prior to death (21, 28). Such enhanced locomotion could arise through modulation of the host clock since the circadian network affects locomotor and sleep output (79). Given that summit disease is closely linked with locomotion and that directionality is indicated by environmental cues such as light and gravity, perhaps fungal effectors target phototaxis or gravitaxis pathways to drive hosts to climb nearby substrates. Work in *O. camponoti-atricipis* has found that infected ants are more likely to be found in well-lit than shaded areas and that the height that infected ants climb before their death varies with the amount of light in their immediate environment (32) This suggests that phototaxis pathways are altered in zombie ants to make them “light seekers.”

So far, our best understanding of pathogen-induced enhanced locomotory activity (ELA) and summiting comes, not from behavior-modulating fungi, but from baculoviruses. In Spodoptera exigua and Bombyx mori, it has been found that the viral protein tyrosine phosphatase (ptp) is essential for baculovirus-elicited ELA (54, 80); in Lymantria dispar, the viral gene ecdysteroid UDP-glucosyltransferase (*egt*) is essential to elicit climbing behavior (81). While the mechanism by which ptp acts is unknown, egt inhibits the molting hormone 20-hydroxyecdysone (20E) (82), which disrupts molting behavior and is hypothesized to cause the host to continue feeding at elevated locations (83). However, follow-up work has found that viral ptp and egt are not necessary for manipulated locomotion and climbing in other baculovirus-caterpillar systems (83, 84). This suggests that a variety of mechanisms are used to achieve these behaviors. Given this diversity of mechanisms seen in the realm of other behavior-modifying pathogens, it seems reasonable to expect that, while parallels might exist, zombifying fungal entomopathogens may also use a variety of mechanisms to elicit host behaviors.

## THE EVOLUTION OF COMMONLY ENCOUNTERED MANIPULATED BEHAVIORS

The ability to manipulate host behavior has convergently evolved in an array of fungal entomopathogens and beyond. This complex trait is thought to be the result of a long, intimate coevolution between pathogens and hosts in which both organisms are in a constant arms race. Despite the independent evolutionary trajectories of each of these pathogens with their respective insect hosts, they have elicited similar types of behaviors. As discussed, fungi across two distinct phyla manipulate host locomotion activity, induce climbing behavior, trigger preference for or attraction to certain abiotic factors, activate mouthparts to adhere to vegetation, and do so with timed precision (Fig. 1). This begs the question: why are these manipulated behaviors so frequently encountered?

One potential answer is that the mechanisms and pathways that lead to these behaviors are simply the easiest to alter. Animals, including insects, can change their behavior as their environment changes. Neuroactive chemicals like hormones and neuromodulators can activate and deactivate behavioral pathways in the central nervous system to allow for quick responses to unpredictable alterations in an individual’s direct surroundings (85). Additionally, behavioral outputs of the biological clock regulate activity patterns to anticipate predictable biotic and abiotic daily changes in the environment, (86, 87) but do so flexibly enough to give rise to interindividual variation within a population (i.e., chronotypes) (88, 89) and adjust to sudden changes in environmental and social cues (90–92). While such behavioral plasticity is of utmost importance for survival, it also provides opportunities for parasites to hijack and coopt (93, 94). Indeed, there is evidence that titers of neuromodulatory biogenic amines, such as serotonin and dopamine, are affected by parasites and pathogens across vertebrate and invertebrate hosts that adaptively manipulate behavior (21, 27, 95–98). Moreover, the clock of *C. floridanus*, the ant host of *O. camponoti-floridani*, appears to be rather plastic and has been suggested to underlie at least some of the plasticity that gives rise to the behavioral division of labor in colonies of this species (99). Reports on the loss of daily activity patterns in *Ophiocordyceps-*infected *C. floridanus* (28), establishment of synchronized manipulated summiting (21), and differential expression of clock and clock-controlled genes in infected individuals compared to healthy conspecifics (21) suggest that behavioral plasticity resulting from biological clocks is corruptible.

Another explanation for the convergent evolution of similar behavioral manipulations could be that these particular behaviors provide such a big advantage in transmission to new hosts that they conferred very strong selection over other altered behaviors. Parasites and pathogens that can alter host behavior are more likely to do so by casting a wide net, instead of selectively attacking discrete areas of nervous tissue (93). As such, a suite of insect host behaviors is likely affected by fungal cells and effectors. Combinations of tissue occupancy and chemical secretions that lead to host phenotypes resulting in higher pathogen fitness will be selected for over time.

In addition, the host’s immune and nervous system are undeniably connected (100–102). Immune responses against invading pathogens result in the release of factors that affect neural function. This, in turn, leads to altered host behaviors that potentially aid in its recovery: so-called sickness behaviors (103–105). Sickness behaviors often involve altered locomotion and feeding activity and diminished interactions with conspecifics. In addition, house flies infected with the generalist *B.* bassiana demonstrate a behavioral thermoregulation response by seeking out hotter and colder temperatures at different times of day (106). Moreover, the nonmanipulating fungus Metarhizium brunneum causes ant hosts to become phototropic and less attracted to social cues, which would eventually result in them straying from the nest (107). As such, infections with nonmanipulating fungi also appear to involve host behavioral changes. However, the interpretation of the function of these behaviors is host adaptive for some (106, 108–110) or pathogen adaptive for others (111), or they are deemed nonfunctional/mere by-products of disease. Nevertheless, generalist entomopathogens may have the tools needed to manipulate, and either we have not observed the behavioral effects in their preferred hosts or their manipulations are much less conspicuous compared to the classic examples of active host transmission and summiting. Regardless of their function, there seems to be a degree of overlap between the common behaviors that behavior-manipulating fungi induce and those that arise from infections with nonmanipulating generalists. This suggests that there could be a sliding scale along which entomopathogenic fungi can evolve with less precise infection-related behaviors on one end and more fine-tuned transmission-benefitting manipulated behaviors on the other.

Considering that behavior is a complex phenotype with multiple underlying mechanisms, the ability to induce a stereotypical set of behaviors at the right time such that it consistently benefits the pathogen’s life cycle is unlikely to evolve quickly. The ability to produce a complex cocktail of compounds that changes behavioral outputs of the brain with exquisite precision requires a long, tight coevolutionary history with the host’s nervous system. Logically, this would come at the cost of promiscuity (Fig. 1), which would explain why only select pathogens have evolved this trait and why those that have are fairly to highly host specific.

The question of why the same manipulated behaviors are so frequently encountered across fungus-insect interactions is currently left unanswered. However, research that would increase our understanding of this topic would be a worthwhile endeavor and is also likely to lead to general insights into the mechanisms underlying behavior plasticity and fungus-insect coevolution.

## CONCLUSIONS

The study of behavior-modifying fungal pathogens is a rapidly growing field, as exemplified by the discovery and (re-)classification of many new species of zombie-making fungi in just the last decade (e.g., references 70 and [Bibr B112][Bibr B113][Bibr B120]). During this time, various -omics and molecular biology techniques have also become more accessible and applicable to nontraditional fungal models. This has allowed for deeper exploration into the complex relationship between zombie-making fungal pathogens and their hosts, resulting in many hypotheses about the underlying mechanisms of host behavior manipulation (Table 1). These hypotheses currently place research on behavior-modifying fungal pathogens at an exciting crossroads toward linking the behavioral phenomes of manipulated insects with the underlying genomes of the fungi that infect them. However, the application of cutting-edge -omics and molecular techniques does not come without challenges. Many zombie-making fungi, especially Entomophthorales, are extremely fastidious, making them difficult to isolate and culture in the lab environment. This complicates the implementation of molecular methods, such as CRISPR-Cas9, and transformation techniques necessary to determine the functional roles of secreted fungal molecules in behavioral manipulation. Work that makes some of these more recalcitrant species lab amenable is, therefore, paramount for continued mechanistic studies. We recognize the difficulties that come along with these endeavors and offer encouragement to researchers who have already or will soon be venturing into this field. With persistence, creativity, and luck, we are optimistic that new zombie-making fungus systems will continue to be harnessed and investigated, as we have seen in recent years.

Future research into the molecular underpinnings of host manipulation is also dependent on a complementary expansion in genomics, transcriptomics, and metabolomics work. For instance, the current relatively low availability of high-quality, annotated genomes, scattered across a few distantly related fungal species, complicates meaningful comparative analyses between these groups. Increasing the number of sequenced genomes within genera and families that harbor at least several zombie-making fungal species would allow for the identification of evolutionary patterns, genes, and gene clusters that give rise to behavior manipulation traits. This, too, however, poses various challenges: where the genomes of hypocrealean Ophiocordycipitaceae and Cordycipitaceae are relatively compact (21.91 to 32.31 Mb for *Ophiocordyceps* species [18, 21]) and straightforward to assemble, entomophthoralean fungi have much larger and repeat-rich genomes (∼1 Gb for *E. muscae* [NCBI accession no. PRJNA479887]), which are assembly averse. The advent of long-read sequencing and ever-improving future technological developments are promising options for solving this issue.

As with any new field of study that ventures forward from descriptive to quantitative research on wild, nontraditional model systems, there are many hurdles to overcome before the mystery of mechanisms underlying fungal manipulation of insect behavior can be fully unraveled. Understanding these pathways will lead to valuable insight into the mechanisms of pathogen-host coevolution and genes underlying host specificity in animal models. Additionally, these efforts will shed new light on how and why animals behave in certain ways and may open the road toward the discovery of novel fungal biomolecules with potential pharmaceutical and industrial applications. Furthermore, characterization of biomolecules secreted by zombie-making fungi and their role in host manipulation at the molecular level will finally allow for a more holistic comparison between these animal pathogens and well-studied host-pathogen interactions in fungus-plant models.
